# Identification of the DNA methylation signature of Mowat-Wilson syndrome

**DOI:** 10.1038/s41431-024-01548-4

**Published:** 2024-02-13

**Authors:** Stefano Giuseppe Caraffi, Liselot van der Laan, Kathleen Rooney, Slavica Trajkova, Roberta Zuntini, Raissa Relator, Sadegheh Haghshenas, Michael A. Levy, Chiara Baldo, Giorgia Mandrile, Carolyn Lauzon, Duccio Maria Cordelli, Ivan Ivanovski, Anna Fetta, Elena Sukarova, Alfredo Brusco, Lisa Pavinato, Verdiana Pullano, Marcella Zollino, Haley McConkey, Marco Tartaglia, Giovanni Battista Ferrero, Bekim Sadikovic, Livia Garavelli

**Affiliations:** 1 Medical Genetics Unit, Azienda USL-IRCCS di Reggio Emilia, 42122 Reggio Emilia, Italy; 2 Department of Human Genetics, Amsterdam Reproduction & Development Research Institute, Amsterdam University Medical Centers, University of Amsterdam, Amsterdam, The Netherlands; 3 Verspeeten Clinical Genome Centre, London Health Science Centre, London, ON Canada; 4 Department of Pathology and Laboratory Medicine, Western University, London, ON Canada; 5 Department of Medical Sciences, University of Torino, Torino, Italy; 6 Molecular Biotechnology Center “Guido Tarone”, University of Torino, 10126 Torino, Italy; 7 Laboratory of Human Genetics, IRCCS Istituto Giannina Gaslini, Genova, Italy; 8 Medical Genetics Unit and Thalassemia Center, San Luigi University Hospital, University of Torino, 10043 Orbassano (Torino), Italy; 9 IRCCS Istituto delle Scienze Neurologiche di Bologna, UOC Neuropsichiatria dell’Età Pediatrica, 40139 Bologna, Italy; 10 Department of Medical and Surgical Sciences (DIMEC), University of Bologna, 40126 Bologna, Italy; 11 Institute of Medical Genetics, University of Zürich, Zürich, Switzerland; 12 Department of Endocrinology and Genetics, University Clinic for Pediatric Diseases, Faculty of Medicine, Ss. Cyril and Methodius University in Skopje, 1000 Skopje, Republic of North Macedonia; 13 Medical Genetics Unit, Città della Salute e della Scienza University Hospital, Torino, Italy; 14 Institute of Genomic Medicine, Department of Life Sciences and Public Health, ‘Sacro Cuore’ Catholic University of Rome, 00168 Rome, Italy; 15 Molecular Genetics and Functional Genomics, Ospedale Pediatrico Bambino Gesù, IRCCS, 00146 Rome, Italy; 16 Department of Clinical and Biological Science, University of Torino, Torino, Italy

**Keywords:** DNA methylation, Neurodevelopmental disorders

## Abstract

Mowat-Wilson syndrome (MOWS) is a rare congenital disease caused by haploinsufficiency of *ZEB2*, encoding a transcription factor required for neurodevelopment. MOWS is characterized by intellectual disability, epilepsy, typical facial phenotype and other anomalies, such as short stature, Hirschsprung disease, brain and heart defects. Despite some recognizable features, MOWS rarity and phenotypic variability may complicate its diagnosis, particularly in the neonatal period. In order to define a novel diagnostic biomarker for MOWS, we determined the genome-wide DNA methylation profile of DNA samples from 29 individuals with confirmed clinical and molecular diagnosis. Through multidimensional scaling and hierarchical clustering analysis, we identified and validated a DNA methylation signature involving 296 differentially methylated probes as part of the broader MOWS DNA methylation profile. The prevalence of hypomethylated CpG sites agrees with the main role of ZEB2 as a transcriptional repressor, while differential methylation within the *ZEB2* locus supports the previously proposed autoregulation ability. Correlation studies compared the MOWS cohort with 56 previously described DNA methylation profiles of other neurodevelopmental disorders, further validating the specificity of this biomarker. In conclusion, MOWS DNA methylation signature is highly sensitive and reproducible, providing a useful tool to facilitate diagnosis.

## Introduction

Mowat-Wilson syndrome (MOWS; OMIM #235730) is a rare neurodevelopmental disorder (NDD) caused by heterozygous deletions or loss-of-function (LoF) variants of the *ZEB2* gene (HGNC:14881; locus 2q22.3) [1, 2]. Affected individuals have a variable phenotype characterized by moderate to severe global developmental delay (DD), microcephaly, and a typical facial appearance including hypertelorism, broad and medially sparse eyebrows, wide nasal bridge with a rounded nasal tip, low hanging columella, pointed chin, and uplifted earlobes with a central depression as major features [3–5]. Eye anomalies often include strabismus and refraction abnormalities. Growth parameters tend to be normal at birth, but drop below the normal range during childhood [6]. More than half of the individuals have chronic constipation, which is mostly caused by Hirschsprung disease (HSCR) [7]. Equally frequent are congenital heart defects (CHD), with a prevalence of septal defects, and abnormalities of the genitourinary system, particularly hypospadias in males. Brain abnormalities at MRI are common, including most prevalently agenesis or hypoplasia of the corpus callosum and morphological or positional anomalies of the hippocampus [8]. Individuals with MOWS show moderate to severe intellectual disability (ID) with relatively good receptive language skills, while expressive language is generally absent or limited to a few words. Nearly all individuals have epilepsy, usually manifesting in the preschool period and presenting with a characteristic, age-related electroclinical pattern [9, 10].

ZEB2 (zinc finger E box-binding homeobox 2; OMIM *605802), also known as ZFHX1B or SIP1 (SMAD-interacting protein 1), is a member of the ZEB family of zinc-finger transcription factors (TFs). It is characterized by a central homeodomain and two clusters of C2H2-type zinc fingers, near the N- and the C-terminus (NZF, CZF), which mediate binding to DNA at E2-box motifs within the regulatory elements of target genes. ZEB2 contributes to the fine-tuning of several cell proliferation and differentiation signals, controlling multiple developmental processes [11]. It can modulate TGFβ/BMP signaling by interacting with SMAD proteins, and has a central role in promoting epithelial to mesenchymal transition (EMT) and cell motility [12].

In vitro and in vivo studies demonstrated that proper spatiotemporal expression of *ZEB2* is essential for correct embryo development. It is highly expressed in neural crest cells (NCCs), inducing EMT and delamination, migration and specification into enteric and peripheral neurons, glial cells, cardiac myocytes, and craniofacial cartilage structures [13]. In the brain, ZEB2 regulates cortical neurogenesis and axonal growth, migration of GABAergic interneurons, and maturation of glial precursors into myelinating oligodendrocytes [14]. Conditional KO animal models have been shown to recapitulate the clinical features of MOWS [13, 15].

ZEB2 is a well-recognized chromatin remodeler. It can act as a transcriptional activator by recruiting histone acetyltransferases (HAT) P300 and KAT2B, but it functions preferentially as a transcriptional repressor. It interacts with proteins of the C-terminal binding (CtBP) family, which downregulate gene expression by recruiting histone deacetylases (HDACs) and methyltransferases (HMTs). The N-terminal region of ZEB2 contains an interaction motif (NIM), capable of binding to the nucleosome remodeling and histone deacetylation (NuRD) co-repressor complex [15, 16]. Of note, in mouse embryonic stem cells (mESCs), Zeb2 was shown to be important in the transcriptional control of *Tet1*, which encodes a DNA methyltransferase with a key role in establishing DNA methylation (DNAm) patterning in the early embryo [17].

Around 350 individuals with molecularly confirmed MOWS have been reported in publications and registries [15], the majority displaying *ZEB2* haploinsufficiency due to intragenic LoF variants or gene deletions. Rare missense variants, usually affecting specific functional domains, have also been reported [16, 18]. These variants generally result in proteins partially retaining ZEB2 function and are associated with milder or atypical MOWS phenotypes, hindering the diagnosis. Notably, the ClinVar database (accessed on August 6^th^, 2023) reports 290 missense variants of uncertain significance (VUS) in the *ZEB2* gene, which attests the difficulty in properly evaluating their functional relevance and clinical significance. On the other hand, some individuals with clinical features fitting MOWS apparently do not show relevant variations in the coding sequence of *ZEB2*, suggesting that variation involving noncoding portions of the gene might account for a proportion of affected individuals. Consistently, recent studies support the presence of proximal and distal noncoding elements implicated in the control of *ZEB2* expression, many of which are still poorly characterized [19].

Constitutive variants in epigenetic regulators, such as HATs, HDACs and HMTs, can determine unique alterations in the DNAm patterns established during embryogenesis [20]. Analysis of these specific alterations, or “episignatures”, using genomic DNA from peripheral blood, is a novel but rapidly growing strategy in the diagnosis of rare mendelian diseases. In particular, the EpiSign classifier has proven to be a useful tool for the reclassification of VUS as well as to confirm/reject a clinical diagnosis [21]. EpiSign v3 assay has been reported to detect over 58 episignatures across more than 65 diseases, in particular NDDs [22–24].

Based on these considerations, we hypothesized that *ZEB2* defects causing MOWS may be associated with a distinctive genome-wide DNAm profile. Here we provide evidence of a DNAm signature for MOWS, based on the analysis of peripheral blood samples from affected individuals with pathogenic variants or deletions of *ZEB2*, offering an informative diagnostic tool for this syndrome.

## Materials and methods

### Study cohort

A total of 29 individuals (17 females and 12 males) were included in the study. We randomly divided the individuals into two different cohorts that were used for the discovery of the episignature (*n* = 24) and its validation (*n* = 5). All individuals had clinical features fitting MOWS and were heterozygous for pathogenic (P)/likely pathogenic (LP) variants or deletions involving *ZEB2*, classified according to the American College of Medical Genetics (ACMG) and Association for Molecular Pathology (AMP) criteria [25, 26].

### DNA methylation profiling

Genomic DNA was extracted from circulating leukocytes. DNAm profiling was performed using the Illumina Infinium MethylationEPIC BeadChip arrays (San Diego, CA, USA), following the manufacturer’s protocol. The resulting intensity data files were loaded into R (version 4.2.3) with minfi (version 1.44.0) [27]. Quality control and feature selection methods, which included normalization, background correction, density plot evaluation, and checking for discrepancies in recorded and predicted age and sex, had previously been reported extensively [28, 29]. We removed probes that overlapped with single-nucleotide variation, cross-reactive probes, probes specific to regions on the X or Y chromosomes, and those with detection *p* value > 0.1 during probe filtering. After this step, 772,557 probes were considered for subsequent analyses.

### DNA methylation data analyses

We conducted DNAm analyses using previously published methods [28, 29]. MatchIt (version 4.5.2) [30] was used to select matched controls from the EpiSign Knowledge Database (EKD) based on sex, age, batch, and array type. Principal component analyses (PCA) were performed to identify potential outliers in the training and matched case-control cohorts. Matched cases and controls underwent feature selection (limma version 3.54.2) [31] and differential methylation analysis was performed using linear regression fitting with the methylation beta values as predictors and methylation labels as response. The model was adjusted for confounding variables, namely estimated blood cell counts. To control for false discoveries, the empirical Bayes method was applied and adjusted using the Benjamini-Hochberg procedure with t-statistics and *p* values. Using different probe sets, we varied the cutoffs for the top *p* values and measured the variable importance through receiver operating characteristic curve analysis and correlation. The clustering between cases and controls was explored using heatmaps and multidimensional scaling (MDS) with ggplots2 (version 3.1.3), and the best clustering was selected. Finally, we conducted leave one-out cross validation and unsupervised clustering to evaluate the reproducibility of the DNAm signature.

### Prediction model

In order to improve the precision of categorizing the case and control samples, we utilized the support vector machine (SMV) algorithm, which was trained through the R package e1071 (version 1.7-13), using the chosen characteristics and the matched controls and cases as training data. To create the classifier, we compared the training samples with the corresponding matched control samples utilized for probe selection, as well as 75% of other controls and samples with known episignatures from the EKD. The remaining 25% of these controls and samples with known episignatures were employed for model testing. A methylation variant pathogenicity (MVP) score ranging from 0 to 1 was generated for each sample, indicating the likelihood of that sample having a methylation profile comparable to that of the MOWS cohort.

### Comparative analysis of DNA methylation data across disease-specific episignatures

Previously published articles were used as a basis for functional annotation and episignature cohort comparison [22–24]. We assessed the percentage of differentially methylated probes (DMPs) shared between the MOWS episignature and those referring to 56 neurodevelopmental disorder episignatures included in the EpiSign v3 clinical classifier, and produced heatmaps and circos plots. The heatmaps were plotted using the R package pheatmap (version 1.0.12), while the circos plots were generated with the R package circlize (version 0.4.15) [32]. We also performed clustering analysis to identify relationships across all 57 cohorts with known episignatures and generated a tree and leaf plot using the R package TreeAndLeaf (version 1.6.1) [33] to visualize the distance and similarities between the cohorts. Additionally, we used the R package annotatr (version 1.20.0) [34] with AnnotationHub (version 3.2.2) as previously described by Levy et al. [24]. to annotate probes in relation to CpG islands (CGIs) and genes and investigate the genomic location of the DMPs characterizing the MOWS cohort.

## Results

### Identification and validation of the MOWS episignature

In the frame of a collaborative project, we collected DNA samples from the peripheral blood of 29 individuals with a clinical diagnosis of MOWS and a pathogenic or likely pathogenic alteration of the *ZEB2* gene or locus. The identified variants were representative of the molecular spectrum of MOWS, including de novo nonsense, frameshift, and missense variants, as well as gene deletions (Table 1). Table 2 summarizes the clinical features of each individual, while a detailed description of the clinical and molecular characteristics is presented in the Supplementary Information.

**Table 1:** Molecular details of the MOWS cohort.

#	Cohort	Age (years) at sample collection	*ZEB2* variant (NM_014795.4, NP_055610.1)	Variant type	ACMG/AMP class (criteria/score)
1	training	4	c.2083 C > T, p.(Arg695*)	Nonsense	5(PVS1,PS2,PM2,PP4)
2	training	8	c.3160 C > G, p.(Pro1054Ala)	Missense	4(PS2,PM1,PM2,PP4)
3	training	9.3	c.2254dup, p.(Thr752Asnfs*4)	Frameshift	5(PVS1,PS2,PM2,PP4)
4	training	9.5	c.310 C > T, p.(Gln104*)	Nonsense	5(PVS1,PS2,PM2,PP1,PP4)
5	training	8.2	c.274 G > T, p.(Gly92*)	Nonsense	5(PVS1,PS2,PM2,PP4)
6	training	2	c.1052del, p.(Gly351Valfs*19)	Frameshift	5(PVS1,PS2,PM2,PP4)
7	training	13.7	c.901del, p.(Leu301Cysfs*37)	Frameshift	5(PVS1,PS2,PM2,PP4)
8	training	12.3	c.625 C > T, p.(Gln209*)	Nonsense	5(PVS1,PS2,PM2,PP4)
9	training	13.6	c.2083 C > T, p.(Arg695*)	Nonsense	5(PVS1,PS2,PM2,PP4)
10	training	8.8	c.477_484del, p.(His159Glnfs*10)	Frameshift	5(PVS1,PS2,PM2,PP4)
11	training	9	c.2701 C > T, p.(Gln901*)	Nonsense	5(PVS1,PS2,PM2,PP4)
12	training	2.3	c.2718del, p.(Ala907Leufs*23)	Frameshift	5(PVS1,PS2,PM2,PP4)
13	training	14.3	c.2180 T > A, p.(Leu727*)	Nonsense	5(PVS1,PS2,PM2,PP4)
14	training	13.6	c.310 C > T, p.(Gln104*)	Nonsense	5(PVS1,PS2,PM2,PP1,PP4)
15	training	11	c.3031del, p.(Ser1011Alafs*64)	Frameshift	5(PS2,PM1,PM2,PM4,PP4)
16	training	3.8	c.1631_1635dup, p.(Asp546Leufs*11)	Frameshift	5(PVS1,PS2,PM2,PP4)
17	training	15.3	c.540del, p.(Glu181Argfs*31)	Frameshift	5(PVS1,PS2,PM2,PP4)
18	training	6.1	c.2682del, p.(Leu894Phefs*36)	Frameshift	5(PVS1,PS2,PM2,PP4)
19	training	6	c.817del, p.(Leu273*)	Nonsense	5(PVS1,PS2,PM2,PP4)
20	training	2.3	arr[hg19] (2q21.1-q22.3)x1 (16.7 Mb, spans *ZEB2*)	Large deletion	pathogenic(1)
21	training	12.4	c.310 C > T, p.(Gln104*)	Nonsense	5(PVS1,PS2,PM2,PP4)
22	training	15.2	c.650_653dup, p.(Gly219Profs*21)	Frameshift	5(PVS1,PS2,PM2,PP4)
23	training	1.4	c.715del, p.(Glu239Argfs*23)	Frameshift	5(PVS1,PM2,PP4)
24	training	3.8	c.1578_1579delinsA, p.(Asp527Thrfs*17)	Frameshift	5(PVS1,PS2,PM2,PP4)
25	validation	4	c.2357dup, p.(Leu786Phefs*9)	Frameshift	5(PVS1,PS2,PM2)
26	validation	10	c.696 C > G, p.(Tyr232*)	Nonsense	5(PVS1,PS2,PM2,PP4)
27	validation	15.7	arr[hg19] (2q22.2-q22.3)x1 (4.6 Mb, spans *ZEB2*)	Large deletion	pathogenic(1)
28	validation	25	c.2083 C > T, p.(Arg695*)	Nonsense	5(PVS1,PS2,PM2,PP4)
29	validation	6.7	c.2717del, p.(Pro906Leufs*24)	Frameshift	5(PVS1,PS2,PM2,PP4)

# sample identifier, *ACMG/AMP* American College of Medical Genetics and Genomics/Association for Molecular Pathology.

**Table 2:** Clinical details of the MOWS cohort.

#	Sex/Age	Craniofacial appearance	Microcephaly	Short stature	DD/ID	Speech	Epilepsy (onset)	Other anomalies						Hearing loss
								Brain MRI	Congenital heart defects	Gastro-intestinal	Genito-urinary	Eye	Tooth	
1	F/2 y	Typical MOWS	+	-	++	absent	+ (12 m)	NA	-	-	-	strabismus, coloboma	-	-
2	F/22 y	Typical MOWS	-	-	++	delay	-	CC, WM	-	-	-	-	+	+
3	M/20 y	Typical MOWS	+	-	++	delay	+ (12 m)	CC, H, WM, LV	ASD, VSD, PDA	HSCR	-	strabismus	+	+
4	F/20 y	Typical MOWS	+	-	+++	absent	+ (11 m)	CC, H	VSD, PDA, CoA, AVS, PS	constipation	+	strabismus	-	-
5	F/19 y	Typical MOWS	-	+	+++	delay	+ (36 m)	LV	-	constipation	-	myopia	+	-
6	F/13 y	Typical MOWS, dolichocephaly	+	-	++	delay	+ (27 m)	H, WM, LV	VSD, PDA, CoA	constipation	-	strabismus, astigmatism	+	-
7	M/24 y	Typical MOWS	+	+	+++	absent	+ (13 m)	H, WM		HSCR	+	nystagmus	+	-
8	M/22 y	Typical MOWS	+	+	+++	NA	+ (36 m)	CC	ASD, PDA, PS, AVS	constipation	-	-	+	+
9	F/23 y	Typical MOWS, turricephaly	+	-	+++	delay	+ (15 m)	CC, H	PDA	constipation	-	nystagmus, astigmatism	+	-
10	F/18 y	Typical MOWS	+	-	+++	delay	+ (6 y)	CC, WM	-	constipation	-	astigmatism	-	-
11	M/18 y	Typical MOWS	+	-	+	delay	+ (12 m)	CC, H, WM	-	constipation	-	-	+	+
12	M/12 y	Typical MOWS, brachycephaly	+	+	++	absent	+ (4 y)	CC, H, LV	VSD, PS, BAV	constipation	+	strabismus	-	-
13	M/22 y	Typical MOWS, dolichocephaly	-	-	+++	absent	+ (9 y)	CC, H, WM	-	-	+	strabismus, astigmatism	+	-
14	F/21 y	Typical MOWS	-	-	+++	delay	+ (18 m)	CC, H, WM, LV	-	-	-	-	-	-
15	F/19 y	Typical MOWS	-	-	+	mild delay (short sentences)	-	CC, H, WM, LV	-	constipation	-	-	+	-
16	F/11 y	Typical MOWS	+	-	+++	delay	+ (9 m)	CC, H	ASD	-	-	strabismus, myopia	+	-
17	F/23 y	Typical MOWS	+	-	+++	absent	+ (24 m)	CC, LV	-	constipation	-	strabismus, nystagmus	+	-
18	F/14 y	Typical MOWS	-	-	+++	delay	+ (30 m)	CC, H	-	-	-		+	-
19	M/14 y	Typical MOWS	+	-	+++	absent	+ (36 m)	CC, H, WM	PDA	HSCR	-	strabismus, myopia	+	-
20	M/10 y	Typical MOWS	+	-	+	absent	+ (5 y)	CC	ASD, VSD, PDA	constipation	+	strabismus	NA	-
21	F/20 y	Typical MOWS	+	-	+++	delay	+ (6 y)	CC, H, WM, LV	ASD	constipation	-	strabismus	+	-
22	F/23 y	Typical MOWS	+	-	+++	absent	+ (1 m)	CC, H, LV	ASD, VSD, PDA	constipation	-	astigmatism	+	-
23	M/8	Typical MOWS	NA	NA	+++	absent	+ (4 y)	CC, H, WM, LV	-	constipation	+	strabismus	NA	NA
24	M/10 y	Typical MOWS, trigonocephaly	+	-	++	delay	+ (4 y)	CC	PDA, PS	-	+	hypermetropia	-	-
25	F/12 y	Typical MOWS	-	-	+++	absent	+ (12 m)	CC, LV	-	-	-	-	+	-
26	F/20 y	Typical MOWS	-	+	+++	delay	+ (31 m)	CC, H, WM, LV	PDA, PS	HSCR	-	strabismus, myopia	+	-
27	F/26 y	Typical MOWS	+	+	+++	absent	+ (14 m)	CC	CoA	-	-	strabismus	+	-
28	M/34 y	Typical MOWS	-	+	+++	delay	+ (16 m)	CC	-	HSCR	+	-	+	-
29	M/13 y	Typical MOWS	-	-	+++	absent	+ (5 y)	H, LV	-	constipation	-	strabismus	-	-

Legend: #, sample identifier; *F* female, *M* male, *y* years, *m* months, *DD/ID* developmental delay/intellectual disability, *MRI* magnetic resonance imaging, *CC* corpus callosum, *H* hippocampus, *WM* white matter, *LV* lateral ventricles, *ASD* atrial septal defect, *VSD* ventricular septal defect, *PDA* patent ductus arteriosus, *PS* pulmonary stenosis, *CoA* coarctation of aorta, *AVS* aortic valve stenosis, *BAV* bicuspid aortic valve, *HSCR* Hirschsprung disease, *NA* not available.

MDS and hierarchical clustering analyses were performed to identify informative probes able to separate individuals with pathogenic variants in *ZEB2* from unaffected individuals, and confirmed the occurrence of a reproducible genomic DNAm profile, supporting the presence of a disease-specific episignature for MOWS. Specifically, we selected 296 differentially methylated CpG probes to train the classifier (Supplementary Table 1), resulting in a clear separation between the MOWS cases (1-24) and unaffected controls. Both unsupervised clustering methods confirmed that, based on differential methylation from the selected probes, our MOWS cases could be reliably distinguished from controls (Supplementary Fig. 1).

**Fig. 1:**
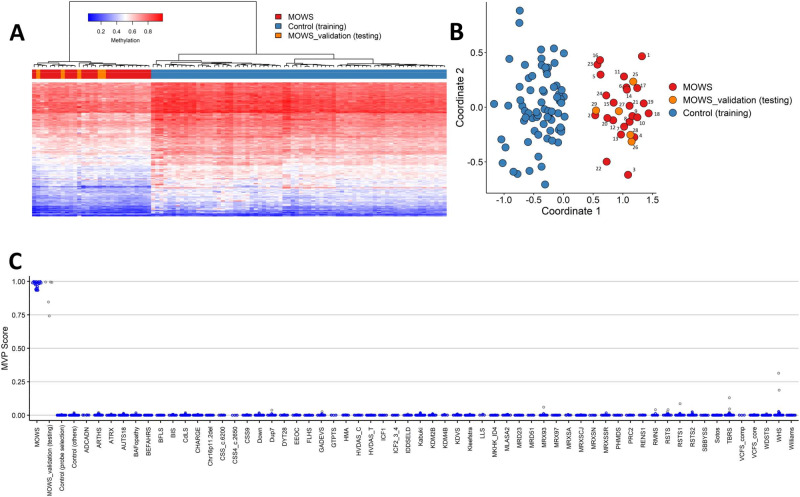
Mowat-Wilson syndrome (MOWS) is characterized by a specific DNAm signature. **A** Euclidean hierarchical clustering heatmap, each column represents one MOWS case or selected control, each row represents one probe selected for this episignature. The heatmap shows a clear separation between cases (in red) and controls (in blue), and properly classifies all validation samples (in orange) with the MOWS cases of the discovery cohort. **B** Multidimensional scaling (MDS) plot shows segregation of MOWS cases and controls. **C** Support Vector Machine (SVM) classifier model. The model was trained using the selected MOWS episignature probes, 75% of controls and 75% of other neurodevelopmental disorder samples (blue). The remaining 25% controls and 25% of other disorder samples were used for testing (grey). Plot shows that all MOWS cases have methylation variant pathogenicity (MVP) scores close to 1.

Next, we performed the validation analysis of the MOWS episignature by assessing a validation cohort of five additional cases with pathogenic variants in *ZEB2* (cases 25–29). Hierarchical clustering and MDS consistently confirmed that all of the tested MOWS cases of this validation cohort clustered with the discovery cohort. The SVM classifier model (MVP score) produced high scores (>0.75) for each of the tested cases, validating the presence of the MOWS-specific DNAm profile (Fig. 1).

Lastly, we performed the final round of training for the MOWS episignature biomarker using the complete set of positive reference controls from the discovery and validation cohort. All 29 MOWS cases clustered together in both heatmap and MDS. Methylation variant pathogenicity analysis resulted in MVP scores close to 1 for all cases, further validating the presence of the MOWS episignature (Supplementary Fig. 2). To test the robustness of this biomarker, 20 rounds of leave-25%-out cross-validation were performed, considering MVP score assessment, unsupervised hierarchical clustering and MDS analysis. Correct classification of all samples was attained, demonstrating robustness, accuracy and specificity (Fig. 1C; Supplementary Fig. 3).

### Overlap of the MOWS genome-wide DNA methylation profile with other disease-specific episignatures

**Fig. 2:**
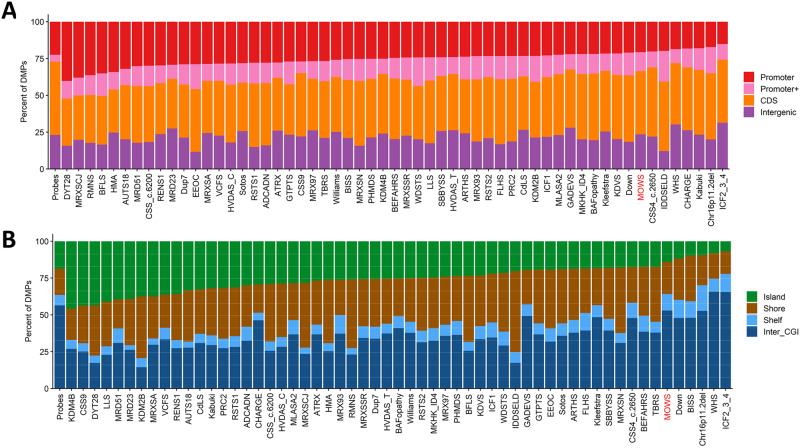
Differentially methylated probes (DMPs) annotated in the context of CpG islands and genes. **A** DMPs in relation to genes. **B** DMPs in CpG islands. Promoter, 0–1 kb upstream of the transcription start site (TSS); Promoter+, 1–5 kb upstream of TSS; CDS, coding sequence; Intergenic, all other regions of the genome. Island, CpG islands; Shore, within 0–2 kb of a CpG island boundary; shelf, within 2–4 kb of a CpG island boundary; Inter_CGI, all other regions in the genome. The “Probes” column in both **A** and **B** represents the background distribution of all array probes determined in the study by Levy et al. [24], considered after initial filtering and used as input for DMP analysis.

To investigate the overlap between the DNAm profiles characterizing the MOWS cohort and those previously obtained for other 56 disorders (EpiSign v3 classifier) [24], we performed functional analysis considering the global DNAm changes occurring in the MOWS cohort. First, we annotated the genomic location of the DMPs in relation to their genomic topological organization. Methylated CpG sites are generally organized in ‘islands’ (CGI), defined as short stretches of DNA (about 500–1500 bp in length) characterized by dense clusters of CpG dinucleotides, that are usually located close to gene promoters. The terms ‘shores’ and ‘shelves’ denote distinct genomic regions with varying CpG densities: ‘shores’ are 2 kb long regions bordering CGIs on both sides, while ‘shelves’ are 2 kb long regions that lie between shores and open genomic areas [24]. 53% of the DMPs were located within Inter_CGI region, 22% in shores, 14% in islands and only 11% in shelves. Of note, no differentially methylated regions (DMRs), defined by at least five consecutive significantly differentially methylated DMPs within 1 kb, were found (Fig. 2).

**Fig. 3:**
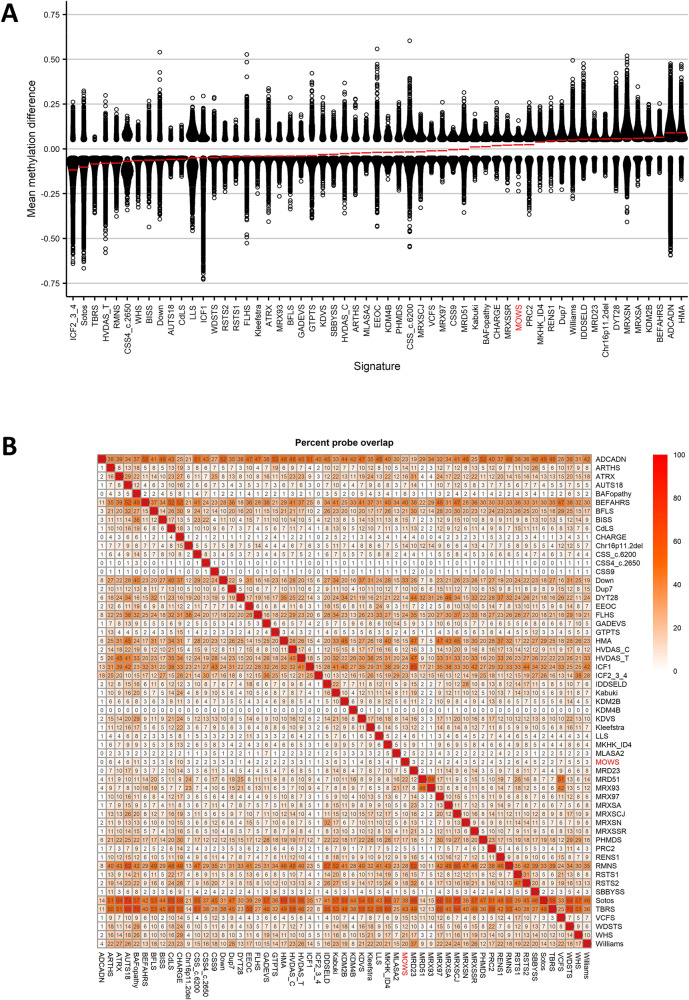
Overlap between the MOWS episignature and the 56 other disorders included in the EpiSign v3 classifier. **A** Global methylation profiles of all differentially methylated probes (DMPs, false discovery rate <0.05) for each cohort, sorted by mean methylation. Each circle represents a single probe, red lines show the mean methylation. **B** Heatmap showing the percentage of probes shared between each paired cohort. Colors indicate the percentage of the y-axis cohort’s probes that are also found in the x-axis cohort’s probes.

Next, we performed comparison analyses to investigate the pattern of DNAm changes between the MOWS episignature and the other 56 episignatures included in the EpiSign v3 classifer [24]. We performed clustering analyses using up to 500 of the most significant DMPs for each cohort. We detected a predominantly hypermethylation profile (Fig. 3A); the highest percentage of overlap in DMPs was with BAFopathies (11%, including *ARID1A*, *ARID1B*, *SMARCB1*, *SMARCA2*, *SMARCA4*), and CHARGE syndrome (10%, *CHD7*) (Fig. 3B and Supplementary Fig. 4).

**Fig. 4:**
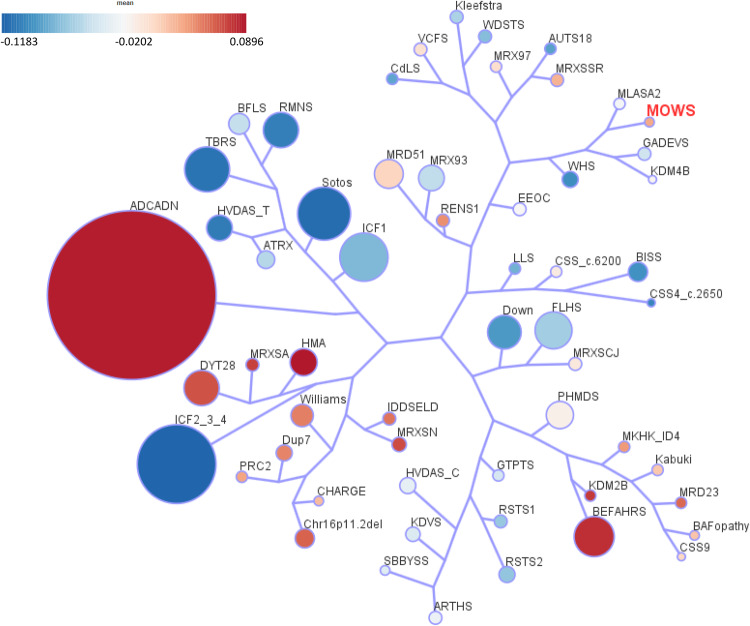
Tree and leaf visualization of Euclidean clustering of all 57 cohorts using the top *n* DMPs for each group, where *n* = min (# of DMPs, 500). Cohort samples were aggregated using the median value of each probe within a group. A leaf node represents a cohort, with node sizes illustrating relative scales of the number of selected DMPs for the corresponding cohort, and node colors are indicative of the global mean methylation difference.

Finally, we also assessed the overall relatedness of the MOWS episignature to the other 56 episignatures described by Levy et al. [24]. MOWS clustered in a branch close to myopathy, lactic acidosis and sideroblastic anemia 2 (MLASA2) caused by pathogenic variants in *YARS2* (Fig. 4).

## Discussion

Disease-specific DNAm signatures provide a valuable biomarker in the diagnosis of rare congenital syndromes [21]. Most NDDs display some degree of phenotypic variability, even in their core neurological features, and episignatures can be highly informative in recognizing the correct syndrome, particularly in the neonatal period or in case of mildly affected individuals with unclear genotyping results.

The aim of this study was to define the genomic DNAm profile and identify an episignature biomarker associated with MOWS. We collected peripheral blood DNA samples from 29 individuals with a confirmed clinical and molecular diagnosis of MOWS. All presented with typical features; two individuals had large genomic deletions including the entire *ZEB2* gene, while 25 had intragenic nonsense or frameshift variants leading to haploinsufficiency. One individual (#15) had a pathogenic frameshift variant in the CZF domain, which was predicted to escape nonsense-mediated mRNA decay, while another (#2) had a likely pathogenic missense variant within the same domain; both had a milder but recognizable clinical phenotype. The classification model was developed using 24 randomly selected samples (including the missense variant and one large deletion) and validated with the remaining five MOWS samples. A final iteration using all 29 samples resulted in the definition of a robust and reproducible episignature based on 296 DMPs, which was highly sensitive and specific for MOWS relative to the DNAm patterns of healthy controls and of other NDDs.

98.6% of the 296 DMPs most relevant to MOWS episignature are hypomethylated (Supplementary Table 1), which is consistent with the main role of ZEB2 as a transcriptional repressor. Although no DMRs were identified, 208 of the episignature DMPs occur within 167 NCBI- or ENSEMBL-annotated genes, and at least one-third map close to transcription start sites (TSS), 5’UTR or first coding exons. According to OMIM, UniProt and NCBI, several of these genes encode TFs and other proteins participating in biological processes matching the known functions of ZEB2 during embryo development, including neuronal development (Supplementary Table 2). Some involve the TGFβ/BMP or Wnt signaling pathways, which are reported to be modulated by ZEB2 to achieve correct spatiotemporal EMT in the development of several tissues [15]. Several DMPs also occur within genes involved in immunity. ZEB2 has a recognized role in hematopoiesis, at least in mouse models, where it is required for the terminal differentiation of dendritic cell, T cell, and natural killer subpopulations, and for the early stage transition from pre-pro-B to pre-B cells [35]. A recent study by Birkhoff et al. cross-referenced ChIP-seq and RNA-seq data generated in mouse models of neural differentiation, and compiled a shortlist of genes that are possibly relevant to the MOWS phenotype and directly regulated by ZEB2 [36]. Interestingly, some DMPs occur within regulatory elements related to the promoters of these genes. They include *GATA3*, encoding a transcriptional activator that is repressed in differentiated T effector cells, in contrast to its presence in T helper 2 cells; *CNTN5* and *CALN1*, respectively a cell-adhesion molecule and a calcium-binding mediator involved in neuron development and physiology; *ZFHX3*, a SMAD-binding TF implicated in myoblast differentiation. The shortlist also includes *RGMB* or Repulsive Guidance Molecule B, a BMP coreceptor involved in the patterning of the developing central and enteric nervous system, while a DMP in this study maps in *RGMA*, which has a similar function but different cell type specification [37].

Sixteen hypomethylated sites, including the four with the most significant *p* value, are located within the *ZEB2* locus itself (Supplementary tables 1 and 3). They are distributed around the TSS and within the second intron, and correspond to the *ZEB2* promoter region and other annotated GeneHancer regulatory elements (Supplementary Fig. 5). Recent in vitro experiments using mESCs have suggested an autoregulatory mechanism, through which Zeb2 appeared to potentiate its own expression to maintain its level sufficiently high during neural differentiation [36]. The occurrence of *ZEB2* hypomethylation, in the setting of the *ZEB2* haploinsufficiency characterizing MOWS samples, may indicate an attempt at compensation by autoregulation. However, caution should be taken in extrapolating the present findings to cell lineages relevant for the developmental and physiological processes implicated in MOWS.

The overlap of MOWS DMPs with other NDDs is very low, indicating a very specific DNAm signature (Fig. 3B), with at most a 10–11% overlap with CHARGE syndrome and BAFopathies. CHARGE syndrome is usually considered in the differential diagnosis of MOWS because of DD/ID associated with CHD, genital hypoplasia and sometimes seizures, but has a distinct ear and facial phenotype, features choanal atresia and does not include HSCR [5]. Its underlying gene, *CHD7*, encodes a transcriptional regulator with helicase activity, expressed in NCC-derived cells at various stages of embryo development [38]. CHD7 and ZEB2 share some cellular pathways, but possibly with different outcomes: in neurogenesis, ZEB2 downregulates pluripotency markers, such as NANOG (directly) and SOX2 (indirectly) [36], while CHD7 acts as a SOX2 cofactor in activating target genes [39]. BAFopathies are a group of NDDs ranging from isolated ID to DD in combination with abnormal morphology of fingers, face and/or hair, which include Coffin-Siris syndrome and Nicolaides-Baraitser syndrome [40]. These syndromes are caused by defects in the components of the BRG1/BRM-associated factor (BAF) chromatin remodeling complexes, also known as SWI/SNF after the yeast homolog. BAF complexes are involved in the transcriptional control of several genes required for cell migration and differentiation, first and foremost in neurogenesis. In particular, some BAF components were demonstrated in vitro to promote EMT and increase *ZEB2* expression in human mammary epithelial cells, suggesting a functional synergy [41].

In the tree-and-leaf representation (Fig. 4) MOWS shares a branch with MLASA2, a metabolic disorder caused by defects in a mitochondrial tRNA synthase encoded by *YARS2* [42]. Apparently there is not much in common between the two conditions or the function of the associated genes. Often the overlap in DMPs is taken to indicate a possible similarity between the underlying biological mechanism of disease, but here it may simply reflect a concordance in the downstream effects of the epigenetic machinery [20]. Interestingly, MOWS and MLASA2 cluster together in a branch with Gabriele-DeVries syndrome (GADEVS) [43] and *KDM4B*-related ID [44]. Individuals with GADEVS are characterized by mild to profound ID with speech delay, and also variable features including craniofacial anomalies (distinct from MOWS), strabismus, skeletal abnormalities of the extremities, feeding difficulties, behavioral issues and rarely seizures [43]. GADEVS is an autosomal dominant disorder caused by de novo variants in *YY1*. Much like *ZEB2*, *YY1* encodes a SMAD-interacting TF featuring two clusters of C2H2-type zinc fingers. It can recruit some of the same repressors or activators including HDAC1/2 and P300, but it has shown a prevalently positive regulation on gene expression in mESC models. It acts downstream of the BMP signaling pathway during early embryogenesis, and has a role in neuronal maturation and function [45]. *KDM4B* encodes a histone demethylase shown in mouse models to be highly expressed during embryo development, especially in the brain [46]. Individuals with heterozygous pathogenic variants display DD with motor and language skills most affected, brain abnormalities, behavioral issues and sometimes seizures. Notably, heterozygous KO mice show hippocampal hypoplasia and corpus callosum agenesis [44], the two most relevant neuroradiological features in MOWS. There is no indication at this point of a direct spatiotemporal co-regulation between *ZEB2* and *YY1* or *KDM4B*, but it is worth noting that they seem to share some common pathways.

In our cohort, the severity of the MOWS phenotype had no apparent correlation with MDS plot clustering and MVP score of the individual samples (Fig. 1, Supplementary Figs. 1–3). In the leave-one-out cross-validation, samples with lower range of MVP score or located in the marginal areas of the cluster in MDS plots did not necessarily correspond to individuals with extreme clinical presentation, either the mildest or the most severe. Similarly, no clear correlation with the type of *ZEB2* variant was noted. For example individual #15, with a C-terminal truncating variant and a milder neurological phenotype (no epilepsy, capable of expressing 3-4 word sentences) [4], clusters near loss-of-function variants associated with severe ID. On the other hand, the two samples with a large locus deletion clustering together with the intragenic variants may be a further indication of *ZEB2* as the main epigenetic machinery-related gene in the region [47].

Missense variants in *ZEB2* have been associated with a variable clinical presentation overlapping with typical MOWS, but often lacking the craniofacial features or other distinctive aspects [16, 18]. One of the samples with a low MVP score corresponds to the only individual with a *ZEB2* missense variant included in this cohort (#2), who also presented with a mild phenotype withouth epilepsy. Although the samples with low MVP score include truncating variants and even a locus deletion, it is tempting to speculate whether pathogenic *ZEB2* missense variants might form a sub-cluster within the MOWS episignature. Analyzing the DNAm signature of further cases will be necessary to test this hypothesis.

In conclusion, we define a specific and reproducible episignature for MOWS as a highly sensitive diagnostic molecular biomarker. The identification of a DNAm signature unlocks the potential of an informative “functional” tool for VUS classification. Functional correlation of genome-wide epigenetic changes provides insight into the molecular mechanisms of *ZEB2* haploinsufficiency, which is expected to guide further research on the molecular pathophysiology of this disorder. Future steps include studies of DNAm patterns of individuals with ambiguous genetic findings and/or atypical clinical presentations of MOWS.

### Supplementary information

Supplementary Information

Supplementary Tables 1-3

## Data Availability

The raw DNA methylation data for the samples are not available due to privacy and ethics restrictions.
