# Supplementary material for: Identification of the DNA methylation signature of Mowat-Wilson syndrome
Source: Eur J Hum Genet. 2024 Feb 13;32(6):619–29. doi: 10.1038/s41431-024-01548-4 (PMC11153515; doi:10.1038/s41431-024-01548-4)
Supplement: Supplementary file 1 — Supplementary Information [file 41431_2024_1548_MOESM1_ESM.pdf]

Identification of the DNA methylation signature of Mowat-Wilson syndrome

Stefano G. Caraffi, Liselot van der Laan, Kathleen Rooney, Slavica Trajkova, Roberta Zuntini, Raissa Relator, Sadegheh Haghshenas, Michael A. Levy, Chiara Baldo, Giorgia Mandrile, Carolyn Lauzon, Duccio Maria Cordelli, Ivan Ivanovski, Anna Fetta, Elena Sukarova, Alfredo Brusco, Lisa Pavinato, Verdiana Pullano, Marcella Zollino, Haley McConkey, Marco Tartaglia, Giovanni Battista Ferrero, Bekim Sadikovic and Livia Garavelli

Supplementary Information

- Supplementary Results: Clinical and Molecular Findings in Our Cohort
- Additional References
- Supplementary Figures 1-5
- Supplementary Tables 1-3 *(separate Excel workbook file)*

Supplementary Results: Clinical and molecular findings in our cohort

(see end of section for additional references)

Individual 1 (not published before) is an 8-year-old female of European ancestry. She is the only affected child in the family born at term from an uneventful pregnancy, during pregnancy which the mother received teratogenic drugs. The proband has typical MOWS facial gestalt, microcephaly, triangular face, wide nasal bridge, bulbous nasal tip, broad flared eyebrows, deep-set eyes, pointed chin, bilateral coloboma, small iris, convergent strabismus, cupped ears with thick lobules, and brittle sparse hair. She presented global developmental delay with sitting at 2 years and walking at around 3 years. She manifested generalized epilepsy from the age of 8 months, not well controlled after treatment with several antiepileptic drugs. She is not verbal and has profound intellectual disability. She has no cardiac anomalies (systolic murmur 2/6) and no gastrointestinal problems. She has a happy demeanor, hand flapping, and stereotypic movements. Chromosomal microarray testing did not reveal deleterious copy number variations; trio exome sequencing identified a heterozygous *de novo* variant in *ZEB2*: NM_014795.4:c.2083C>T, p.(Arg695*). This variant, expected to undergo nonsense-mediated mRNA decay or to result in a truncated protein, was classified as pathogenic according to the recommendations of the American College of Genetics and Genomics/Association of Molecular Pathology (ACMG/AMP; Richards et al., 2015 [25], criteria PVS1, PS2, PM2, PP4).

Individual 2 (not published before) is a 22-year-old female, born from an uneventful pregnancy with spontaneous delivery at 41 gestational weeks. She showed normal neonatal and motor development. The language was impaired, with a tested total IQ of 78 (WPPSI scale). She never had seizures and the EEG was normal. Brain MRI showed hypoplasia of corpus callosum and bilateral gliotic areas in the peritrigonal area. At 5 years of age her measurements were weight 20 kg (50th centile), height 110 cm (50th centile), head circumference 51.5 cm (75th centile). In addition, she presented with mild scoliosis, frequent upper airways infections, and she underwent adenoidectomy. She showed some dysmorphic features, including supernumerary nipples, widely spaced teeth, median flare of the eyebrows, wide nasal bridge and prominent columella. Array CGH testing was normal; trio exome sequencing identified a heterozygous *de novo* missense variant in *ZEB2*: NM_014795.4:c.3160C>G; p.(Pro1054Ala). This variant occurs within the critical C-terminal zinc finger region, known to harbor other pathogenic missense variants (Ghoumid et al., 2013 [18]), and was classified as likely pathogenic (ACMG/AMP criteria PS2, PM1, PM2, PP1, PP4).

Individual 3 (described as patient #17 in Garavelli et al., 2009 [48], #8 in Cordelli et al., 2013 [10], #40 in Garavelli et al., 2017 [8] and Ivanovski et al., 2018 [4], #20 in Ricci et al., 2021 [9]) is currently a 20-year-old male. He has a typical Mowat-Wilson syndrome (MOWS) facial gestalt. Psychomotor development: sitting at 18 months. Standing with support at 24 months. Independent walking at 3 years. Absent language. He was operated for bowel obstruction due to Hirschsprung's disease, confirmed by biopsy, and underwent cardiac surgery for PDA, ventricular defect, ostium secundum atrial septal defect. He underwent cochlear implantation for bilateral sensorineural hearing loss. He had seizures with onset at the age of one year, initially in the course of fever and then in apyrexia. Valgus osteotomy surgery of the calcaneus was performed at 16 years old. Brain MRI examinations showed hypoplasia of the splenium of the corpus callosum with reduction of the corpus callosum also in its posterior third. Molecular analysis MLPA and Sanger sequencing) of the *ZEB2* gene revealed *de novo* heterozygous variant NM_014795.4:c.2254dup, p.(Thr752Asnfs*4), classified as pathogenic (ACMG/AMP criteria PVS1, PS2, PM2, PP4).

Individual 4 (described as patient #2 in Cecconi et al., 2008 [49], #6 in Garavelli et al., 2009 [48], #11 in Cordelli et al., 2013 [10], #30 in Garavelli et al., 2017 [8] and Ivanovski et al., 2018 [4], #31 in Ricci et al., 2021 [9]) is currently a 20-year-old female. She is the sister of individual #14. At 3 days of life she was diagnosed with congenital heart disease with patent foramen ovale, multiple ventricular septal defects, aortic valve stenosis,

aortic coarctation, pulmonic valve stenosis for which she underwent corrective surgery at 27 days of life. Her clinical conditions worsened as a result of pulmonary infectious complications, cardiocirculatory arrest, cerebral hypoxic suffering resulting in flaccid paraplegia and neurological bladder. At the age of 11 months, she underwent an operation of partial correction of the scoliotic and lordotic deformity and of the pelvic obliquity with instrumented posterior vertebral arthrodesis with anchorage to the sacrum. She has a typical MOWS facial gestalt. She has global developmental delay with no speech. Molecular analysis of the *ZEB2* gene gave the following result: *de novo* heterozygous nonsense variant NM_014795.4:c.310C>T, p.(Gln104*), classified as pathogenic (ACMG/AMP criteria PVS1, PS2, PM2, PP1, PP4). The same variant was found in the affected sister, but not in the healthy parents, compatible with germinal mosaicism.

Individual 5 (described as patient #4 in Garavelli et al., 2009 [48], #4 in Cordelli et al., 2013 [10], #20 in Garavelli et al., 2017 [8] and Ivanovski et al., 2018 [4], #22 in Ricci et al., 2021 [9]) is a 19-year-old female. She has typical MOWS facial features. She presented with psychomotor developmental delay with independent walking at 4 years and first words at 4 years. She was diagnosed with severe intellectual disability and epilepsy with onset of generalized seizures at 4 years and absence seizures at 8 years. She is under ophthalmological follow-up for myopia and physiatric-orthopaedic for pectus excavatum, genu valgus, pes planus. Brain MRI revealed an enlargement of the temporal horns of the lateral ventricles. Molecular analysis of the *ZEB2* gene gave the following result: *de novo* heterozygous nonsense variant NM_014795.4:c.274G>T, p.(Gly92*), classified as pathogenic (ACMG/AMP criteria PVS1, PS2, PM2, PP4).

Individual 6 (described as patient #5 in Garavelli et al., 2017 [8] and Ivanovski et al., 2018 [4], #16 in Ricci et al., 2021 [9]) is a 13-year-old female. She has typical MOWS facial gestalt. She had a diagnosis of congenital heart disease (patent ductus arteriosus, ventricular septal defect and aortic coarctation) She presented with psychomotor developmental delay with independent walking at 3 years and 7 months, first words at 3 years and 6 months and moderate intellectual disability. She was diagnosed with epilepsy with onset of focal clonic seizures at 2 years and 3 months and absence seizures at 7 years. She is under ophthalmological follow-up for strabismus and astigmatism and she has pes planus and genu valgus. Molecular analysis of the *ZEB2* gene gave the following result: *de novo* heterozygous frameshift variant NM_014795.4:c.1052del, p.(Gly351Valfs*19), classified as pathogenic (ACMG/AMP criteria PVS1, PS2, PM2, PP4).

Individual 7 (described as patient #1 in Garavelli et al., 2005 [50], #12 in Garavelli et al., 2009 [48], #17 in Cordelli et al., 2013 [10], #47 in Garavelli et al., 2017 [8] and Ivanovski et al., 2018 [4], #35 in Ricci et al., 2021 [9]) is a 24-year-old male. He underwent surgery at age 50 days for Hirschsprung's disease. He had hypospadias and underwent surgery for right cryptorchidism. He has a typical MOWS facial gestalt. Psychomotor development: sitting at 36 months. He has not achieved independent walking. Absent language. He has a severe intellectual disability. He had seizures with onset at the age of 13 months, initially in the course of fever and then in apyrexia. He has epilepsy with focal seizures with secondary generalization. Pediatric neurologists diagnosed complex partial epilepsy and absence seizures. At the age of 13 years, pediatric nephrologists diagnosed kidney stones. He was diagnosed with chronic iron deficiency anemia and presented episodes of intestinal subocclusion. Molecular analysis of the *ZEB2* gene gave the following result: *de novo* heterozygous frameshift variant NM_014795.4:c.901del, p.(Leu301Cysfs*37), classified as pathogenic (ACMG/AMP criteria PVS1, PS2, PM2, PP4).

Individual 8 (described as patient #16 in Cordelli et al., 2013 [10], #26 in Garavelli et al., 2017 [8] and Ivanovski et al., 2018 [4], #33 in Ricci et al., 2021 [9]) is a 22-year-old male with a typical MOWS-evoking facial phenotype. He has congenital heart disease: patent ductus arteriosus, atrial septal defect, pulmonary valve and peripheral stenosis and aortic valve stenosis. Also, he has webbed penis and skeletal involvement: pectus carinatum, scoliosis, genu valgus, pes planus calcaneovalgus. He has sensorineural hearing loss and intellectual disability. He has epilepsy with onset at 3 years, seizures begin during sleepiness and wakes staring, has focal seizures with secondary generalization. Brain MRI showed corpus callosum hypoplasia, lateral ventricles enlargement and

hippocampal malrotation. Molecular analysis of the gene *ZEB2* demonstrated the *de novo* heterozygous nonsense variant NM_014795.4:c.625C>T, p.(Gln209*), classified as pathogenic (ACMG/AMP criteria PVS1, PS2, PM2, PP4).

Individual 9 (described as patient #14 in Garavelli et al., 2009 [48], #18 in Cordelli et al., 2013 [10] and #58 in Ivanovski et al., 2018 [4], #27 in Ricci et al., 2021 [9]) is a 23-year-old female. She has typical MOWS facial gestalt. At the age of 3 months she underwent cardiac surgery for patent Botallo duct. She has hypotonia, motor delay with acquisition of sitting at 5 years, first words at 7 years; she is unable to walk and has a limited language of about ten words, with severe intellectual disability. She had seizures with an onset at 18 months and a previous diagnosis of myoclonic epilepsy, with a tendency to generalize and improve over the years. Brain MRI demonstrated agenesis of the corpus callosum, dilatation of the temporal horns of the lateral ventricles, and small heterotopic subependymal gray matter nodule along the superior contour of the body of the left lateral ventricle. Molecular analysis of the gene *ZEB2* demonstrated the *de novo* heterozygous nonsense variant NM_014795.4:c.2083C>T, p.(Arg695*), classified as pathogenic (ACMG/AMP criteria PVS1, PS2, PM2, PP4).

Individual 10 (described as patient #5 in Cordelli et al., 2013 [10], #57 in Ivanovski et al., 2018 [4], #21 in Ricci et al., 2021 [9]) is a 18-year-old female. She has typical MOWS facial gestalt. Psychomotor development: she acquired the sitting position at 18 months and independent walking at 3 yearst, first words at 4 years. Because of the language delay she uses sign language. At the age of 5 years and 7 months she started having seizures in apyrexia, for which she was diagnosed with epilepsy, with focal seizures and absences. Echocardiogram demonstrated mild pulmonary valve stenosis, while brain MRI showed a thin corpus callosum and dehiscence of the superior semicircular canal. Molecular analysis of the gene *ZEB2* demonstrated the *de novo* heterozygous frameshift variant NM_014795.4:c.477_484del, p.(His159Glnfs*10), classified as pathogenic (ACMG/AMP criteria PVS1, PS2, PM2, PP4).

Individual 11 (described as patient #7 in Cordelli et al., 2013 [10], #31 in Garavelli et al., 2017 [8] and Ivanovski et al., 2018 [4], #23 in Ricci et al., 2021 [9]) is a 18-year-old male. His facial features are compatible with those of the MOWS; he also has superior pectus carinatum/inferior pectus excavatum. Psychomotor development: first words at 18 months, he acquired the independent walking at 2 years. He had seizures with onset at the age of one year, initially during fever and then, at the age of 6 years, in apyrexia. He has had focal seizures with secondary generalization and then, since age 8, absence seizures. Brain MRI demonstrated corpus callosum hypoplasia and hippocampal malrotation. He had recurrent otitis media and was diagnosed with conductive hearing loss. Molecular analysis of the gene *ZEB2* demonstrated the *de novo* heterozygous nonsense variant NM_014795.4:c.2701C>T, p.(Gln901*), classified as pathogenic (ACMG/AMP criteria PVS1, PS2, PM2, PP4).

Individual 12 (described as patient #34 in Garavelli et al., 2017 [8] and Ivanovski et al., 2018 [4], #13 in Ricci et al., 2021 [9]) is a 12-year-old male. In the first month of his life and then at the age of 4 years he underwent cardiac surgery for congenital heart disease with pulmonary atresia, ventricular septal defect and bicuspid aortic valve. He has typical MOWS facial gestalt. Psychomotor development: he acquired the sitting position at 9 months and independent walking at 2 years, he has language delay. He was diagnosed with grade 2 hypospadias and was operated on for bilateral cryptorchidism. He had his first seizure at the age of 4 years with focal seizure with secondary generalization. Brain MRI showed corpus callosum agenesis, hippocampal malrotation and large basal ganglia. Molecular analysis of the gene *ZEB2* demonstrated the *de novo* heterozygous frameshift variant NM_014795.4:c.2718del, p.(Ala907Leufs*23), classified as pathogenic (ACMG/AMP criteria PVS1, PS2, PM2, PP4).

Individual 13 (described as patient #2 in Garavelli et al., 2005 [50], #16 in Garavelli et al., 2009 [48], #14 in Cordelli et al., 2013 [10], #11 in Garavelli et al., 2017 [8] and Ivanovski et al., 2018 [4], #28 in Ricci et al., 2021 [9]) is a 22-year-old male. He has typical MOWS facial gestalt. Psychomotor development: he acquired the sitting position

at 12 months and independent walking at 23 months, he has language delay. He underwent surgery for hypospadias with penoscrotal meatus and severe chorda penis. He has scoliosis and hallux valgus. At the age of 9 years he presented the 1st convulsive episode in apyrexia with generalized crises. Brain MRI showed ectopia of the cerebellar tonsils below the foramen magnum (Chiari 1 malformation) and corpus callosum thinned posteriorly. Molecular analysis of the gene *ZEB2* demonstrated the *de novo* heterozygous nonsense variant NM_014795.4:c.2180T>A, p.(Leu727*), classified as pathogenic (ACMG/AMP criteria PVS1, PS2, PM2, PP4).

Individual 14 (described as patient #1 in Cecconi et al., 2008 [49], #5 in Garavelli et al., 2009 [48], #12 in Cordelli et al., 2013 [10], #29 in Garavelli et al., 2017 [8] and Ivanovski et al., 2018 [4], #34 in Ricci et al., 2021 [9]) is a 21-year-old female. She is the sister of individual #2. She has a typical MOWS facial gestalt. She had delayed psychomotor development with independent walking at two and a half years, first words at 5 years. She had seizures with onset at the age of 18 months, initially in the course of fever and then in apyrexia, with absence, myoclonic and tonic-clonic seizures. Brain MRI examinations showed agenesis of the corpus callosum. Molecular analysis of the *ZEB2* gene gave the following result: *de novo* heterozygous nonsense variant NM_014795.4:c.310C>T, p.(Gln104*), classified as pathogenic (ACMG/AMP criteria PVS1, PS2, PM2, PP1, PP4). The same variant was found in the affected sister, but not in the healthy parents, compatible with germinal mosaicism.

Individual 15 (described as patient #6 in Cordelli et al., 2013 [10], #23 in Garavelli et al., 2017 [8] and Ivanovski et al., 2018 [4], #9 in Ricci et al., 2021 [9]) is a 19-year-old girl of Italian origin with typical MOWS facial gestalt and some EEG anomalies (activation of spikes and waves during sleep). No epileptic seizures have been reported so far. MRI examinations of the brain showed a complete corpus callosum agenesis, lateral ventricles enlargement, hippocampal malrotation, reduction of white matter thickness and large basal ganglia. The patient has a mild intellectual disability and was able to pronounce her first words at 18 months. Currently she can construct simple sentences of 3 to 4 words. She was able to sit without support at 10 months and walked without help at 20 months of age. She has constipation, but no cardiac, urogenital, dental, or skeletal anomalies. Gene sequencing of *ZEB2* identified a *de novo* heterozygous single base pair deletion in exon 9, NM_014795.4:c.3031del, p.(Ser1011Alafs*64), which is predicted to escape nonsense-mediated decay (https://nmdprediction.shinyapps.io/nmdescpredictor/) and result in a truncated protein missing the C-terminal zinc finger cluster, CZF. The variant was classified as pathogenic (ACMG/AMP criteria PS2, PM1, PM2, PM4, PP4).

Individual 16 (described as patient #28 in Garavelli et al., 2017 [8] and Ivanovski et al., 2018 [4]) is a female who died suddenly at the age of 10 years and 10 months. She had the typical MOWS facial features. She had a congenital heart disease, atrial septal defect. Psychomotor development: she acquired the sitting position at 24 months and independent walking at 3 years, she had language delay. She had epilepsy with onset of myoclonic seizures at 9 months. She had recurrent otitis media and was in ophthalmological follow-up for strabismus and myopia. Brain MRI demonstrated corpus callosum hypoplasia and hippocampal malrotation. Molecular analysis of the *ZEB2* gene gave the following result: *de novo* heterozygous frameshift variant NM_014795.4:c.1631_1635dup, p.(Asp546Leufs*11), classified as pathogenic (ACMG/AMP criteria PVS1, PS2, PM2, PP4).

Individual 17 (described as patient #8 in Garavelli et al., 2009 [48], #13 in Cordelli et al., 2013 [10] and #55 in Ivanovski et al., 2018 [4], #32 in Ricci et al., 2021 [9]) is a 23-year-old girl with typical MOWS facial gestalt. She has seizures with onset at the age of 2 years, initially in the course of fever and then, at the age of 6 years, in apyrexia. She has focal seizures with secondary generalization. She is in in ophthalmological follow-up for strabismus and astigmatism. Brain MRI showed thin corpus callosum and lateral ventricles enlargement. Molecular analysis of the *ZEB2* gene gave the following result: *de novo* heterozygous frameshift variant NM_014795.4:c.540del, p.(Glu181Argfs*31), classified as pathogenic (ACMG/AMP criteria PVS1, PS2, PM2, PP4).

Individual 18 (described as patient #2 in Cordelli et al., 2013 [10], #19 in Garavelli et al., 2017 [8] and Ivanovski et al., 2018 [4], #15 in Ricci et al., 2021 [9]) is a 14-year-old girl with typical MOWS facial gestalt. She has pes planus and genu valgus. Psychomotor development: she acquired the sitting position at 10 months and independent walking at 3 years, she spoke her first words at age 2 and had speech delay. She has epilepsy with onset of focal seizures and absence seizures at 2 years and 6 months. Brain MRI demonstrated complete agenesis of corpus callosum and hippocampal malrotation. Molecular analysis of the *ZEB2* gene gave the following result: de novo heterozygous frameshift variant NM_014795.4:c.2682del, p.(Leu894Phefs*36), classified as pathogenic (ACMG/AMP criteria PVS1, PS2, PM2, PP4).

Individual 19 (described as patient #27 in Garavelli et al., 2017 [8] and Ivanovski et al., 2018 [4]) is a 14-year-old boy with typical MOWS facial gestalt. He acquired sitting by age 2, had not acquired independent walking at the age of 8 years, and speech was absent. He has a moderate-severe intellectual disability. He had onset of epilepsy at the age of 3 years with generalized tonic-clonic seizures. He was diagnosed with patent ductus arteriosus, pyloric stenosis, Hirschsprung disease and cryptorchidism. He is in in ophthalmological follow-up for strabismus, myopia and astigmatism. Brain MRI showed partial agenesis of corpus callosum, ventricular temporal horn enlargement and hippocampal malrotation. Molecular analysis of the *ZEB2* gene gave the following result: *de novo* heterozygous nonsense variant NM_014795.4:c.817del, p.(Leu273*), classified as pathogenic (ACMG/AMP criteria PVS1, PS2, PM2, PP4).

Individual 20 (described as patient #61 in Ivanovski et al., 2018 [4], #8 in Ricci et al., 2021 [9]) is a 10-year-old boy with typical MOWS facial gestalt. He was diagnosed with congenital heart disease (patent ductus arteriosus, atrial septal defect, ventricular septal defect), hypospadias and cryptorchidism. He is in ophthalmological follow-up for strabismus. He had onset of focal seizures at the age of 5 years. Brain MRI showed corpus callosum hypoplasia and hippocampal malrotation. Cytogenetic analysis demonstrated a *de novo* heterozygous 16.7Mb deletion encompassing the *ZEB2* gene: (2q21.1-q22.3)x1, classified as pathogenic according to ACMG/ClinGen recommendations for copy number variants (Riggs et al., 2020 [26]; score 1).

Individual 21 (described as patient #7 in Garavelli et al., 2009 [48], #8 in Garavelli et al., 2017 [8] and Ivanovski et al., 2018 [4], #25 in Ricci et al., 2021 [9]) is a 20-year-old girl with typical MOWS facial gestalt. Echocardiography demonstrated an atrial septal defect, while renal ultrasonography showed bilateral pyelic dilatation. She acquired sitting by age 12 months, acquired independent walking after 5 years, first words at 6 years. She had onset of epilepsy at the age of 6 years, with focal clonic seizures. She was in orthopedic follow-up for bilateral pes planus calcaneovalgus and in ophthalmological follow-up for strabismus. Brain MRI showed complete agenesis of corpus callosum, ventricular temporal horn enlargement and hippocampal malrotation. Molecular analysis of the *ZEB2* gene gave the following result: *de novo* heterozygous nonsense variant NM_014795.4:c.310C>T, p.(Gln104*), classified as pathogenic (ACMG/AMP criteria PVS1, PS2, PM2, PP4).

Individual 22 (described as patient #19 in Cordelli et al., 2013 [10], #62 in Ivanovski et al., 2018 [4], #17 in Ricci et al., 2021 [9]) is a 23-year-old girl with typical MOWS facial gestalt. At 10 months of age, she underwent cardiac surgery for atrial septal defect, ventricular septal defect, and patent ductus arteriosus. At 40 days of life, the first convulsive crisis in apyrexia appeared, which was followed by other convulsive episodes. A diagnosis of drug-resistant epilepsy with complex partial seizures with secondary generalization and absence seizures was made. Psychomotor development: she acquired sitting position at 12 months, acquired independent walking at 2 years and 6 months, absent speech. Brain MRI showed complete agenesis of corpus callosum and lateral ventricles enlargement. Molecular analysis of the *ZEB2* gene gave the following result: *de novo* heterozygous frameshift variant NM_014795.4:c.650_653dup, p.(Gly219Profs*21), classified as pathogenic (ACMG/AMP criteria PVS1, PS2, PM2, PP4).

Individual 23 (described as patient #87 in Ivanovski et al., 2018 [4], #1 in Ricci et al., 2021 [9]) is an 8-year-old male of Ukrainian nationality with typical MOWS facial gestalt. The psychomotor development delay (head control acquired at 1 year) and the presence of typical features prompted a neurological and genetic evaluation in Italy at 1 year and 4 months, ultimately leading to genetic testing that confirmed the clinical suspicion.
At the time of the first evaluation, the proband also displayed obstinate constipation and renal involvement. Moreover, he underwent surgery for cryptorchidism. The echocardiogram ruled out the presence of cardiac anomalies. At the age of 4 years he presented with secondarily generalized focal seizures for which he initiated treatment with valproic acid, with partial benefit. Currently, the child has no speech and his motor condition has deteriorated due to the impossibility to access rehabilitative treatments in his home country. During the past three years he has lost the ability to maintain an upright position, which he had previously acquired. Molecular analysis of the *ZEB2* gene gave the following result: heterozygous frameshift variant NM_014795.4:c.715del, p.(Glu239Argfs*23), classified as pathogenic (ACMG/AMP criteria PVS1, PM2, PP4). Parents were unavailable for segregation analysis.

Individual 24 (described as patient #89 in Ivanovski et al., 2018 [4]) is a 10-year-old male with typical MOWS facial gestalt. Echocardiography demonstrated patent ductus arteriosus, muscular interventricular septal defect and pulmonary valve dysplasia. At the age of 4 months he underwent decompressive craniotomy and bilateral fronto-orbital cranioplasty for craniostenosis (trigonocephaly). At the age of 1 year and 6 months he underwent surgery for hypospadias. Psychomotor development: he acquired the sitting position at 9 months, acquired independent walking at 16 months and presented language delay. At the age of 3 years and 6 months, he began having febrile seizure episodes. Molecular analysis of the *ZEB2* gene gave the following result: *de novo* heterozygous frameshift variant NM_014795.4:c.1578_1579delinsA, p.(Asp527Thrfs*17), classified as pathogenic (ACMG/AMP criteria PVS1, PS2, PM2, PP4).

Individual 25 (not published before) is a 12-year-old female who has typical MOWS facial features. Agenesis of the corpus callosum and enlarged lateral ventricles were diagnosed prenatally and confirmed after birth. She presented with epilepsy at 12 months of age, consistent with neurodevelopmental delay. She had two episodes of febrile seizures and subsequently developed tonic-clonic seizures during sleep; she is currently being treated with valproate and levetiracetam and had no further episodes since then. She started walking at 3 years of age, showing ataxic and broad-based gait, flat support with bilateral valgopronation, valgus of the first ray on the left, both knees kept flexed for a few degrees in all phases of the step. Neurodevelopmental delay became more pronounced during the second year of life (Griffiths score 33 at 2 years, 23 at 5 years). At 5 years she was diagnosed with dorsal lumbar scoliosis, treated with Milwaukee brace. No relevant visual/hearing impairment was noted. Molecular analysis of the *ZEB2* gene gave the following result: *de novo* heterozygous nonsense variant NM_014795.4:c.2357dup, p.(Leu786Phefs*9), classified as pathogenic (ACMG/AMP criteria PVS1, PS2, PM2).

Individual 26 (described as patient #10 in Garavelli et al., 2009 [48], #9 in Cordelli et al., 2013 [10], #6 in Garavelli et al., 2017 [8] and Ivanovski et al., 2018 [4], #24 in Ricci et al., 2021 [9]) is a 20-year-old girl with typical MOWS facial gestalt.. She was diagnosed with complex congenital heart disease: pulmonary artery sling with tracheal stenosis, pulmonary valvular and peripheral stenosis, patent ductus arteriosus. She was also diagnosed with Hirschsprung disease and spleen agenesis. Psychomotor development: he acquired the sitting position at 17 months, acquired independent walking at the age of 6 years and presented language delay. She had onset of epilepsy at the age of 2 years and 7 months with myoclonic epilepsy with sleep seizures. She was in orthopedic follow-up for bilateral pes planus calcaneovalgus and in ophthalmological follow-up for strabismus, myopia and astigmatism. Brain MRI showed hypoplasia of the splenium of the corpus callosum, ventricular temporal horn enlargement and hippocampal malrotation. Molecular analysis of the *ZEB2* gene gave the following result: *de novo* heterozygous nonsense variant NM_014795.4:c.696C>G, p.(Tyr232*), classified as pathogenic (ACMG/AMP criteria PVS1, PS2, PM2, PP4).

Individual 27 (described as patient #1 in Garavelli et al., 2009 [48], #20 in Cordelli et al., 2013 [10], #18 in Garavelli et al., 2017 [8] and Ivanovski et al., 2018 [4]) is a 26-year-old girl with typical MOWS facial gestalt. At the age of 2 years she underwent isthmic angioplasty for aortic coarctation. Psychomotor development: she acquired the sitting position at 12 months. She stands with support with left foot equinus-varus and out-rotated. Absent speech. Appearance of seizures with fever at the age of 15 months (myoclonic seizures), subsequently the seizures changed and became "absence" type. She has been in ophthalmological follow-up for exotropia, and is in ENT-audiometric follow-up for severe bilateral sensorineural hearing loss. She also is in orthopedic follow-up for scoliosis and genu valgus. Brain MRI showed hypoplasia of the splenium of the corpus callosum. Array-CGH analysis demonstrated a *de novo* heterozygous 4.6-Mb deletion encompassing the *ZEB2* gene: arr[GRCh37] 2q22.2q22.3(143583530x2, 143743530_148043530x1, 148483530x2) dn, classified as pathogenic according to ACMG/ClinGen recommendations for copy number variants (score 1).

Individual 28 (described as patient #1 in Cerruti-Mainardi et al., 2004 [51], #3 in Garavelli et al., 2005 [50], #15 in Garavelli et al., 2009 [48], #22 in Cordelli et al., 2013 [10], #32 in Garavelli et al., 2017 [8] and Ivanovski et al., 2018 [4]) is a 34-year-old male with typical MOWS facial gestalt. He was diagnosed with Hirschsprung disease, hypospadias, cryptorchidism, and vesicoureteral reflux. He has seizures with onset at the age of 16 months, initially during fever and then, at the age of 3 years, in apyrexia. He has focal seizures with secondary generalization and absence seizures. He was in orthopedic follow-up for bilateral pes planus calcaneovalgus. Psychomotor development: he acquired the sitting position at the age of 3 years, acquired independent walking at the age of 6 years and presented language delay. Brain MRI showed hypoplasia of the corpus callosum, which is thin. Molecular analysis of the *ZEB2* gene gave the following result: *de novo* heterozygous nonsense variant NM_014795.4:c.2083C>T, p.(Arg695*), classified as pathogenic (ACMG/AMP criteria PVS1, PS2, PM2, PP4).

Individual 29 (described as patient #53 in Garavelli et al., 2017 [8] and Ivanovski et al., 2018 [4]) is a 13-year-old male. He has a typical MOWS facial gestalt and has been affected by various types of epileptic seizures (absence, focal with secondary generalization, myoclonic and generalized tonic-clonic). Brain MRI examinations showed lateral ventricles enlargement and hippocampal malrotation. He has severe intellectual disability and absent speech. At the age of 4 years he was able to sit without support and walked without support at 5.5 years old. He has constipation and strabismus, but no cardiac, urogenital, dental or skeletal anomalies. Molecular analysis of the *ZEB2* gene gave the following result: *de novo* heterozygous frameshift variant NM_014795.4:c.2717del, p.(Pro906Leufs*24), classified as pathogenic (ACMG/AMP criteria PVS1, PS2, PM2, PP4).

Additional references:

48. Garavelli L, Zollino M, Mainardi PC, Gurrieri F, Rivieri F, Soli F, et al. Mowat-Wilson syndrome: facial phenotype changing with age: study of 19 Italian patients and review of the literature. Am J Med Genet A. 2009 Mar;149A(3):417–26.
49. Cecconi M, Forzano F, Garavelli L, Pantaleoni C, Grasso M, Dagna Bricarelli F, et al. Recurrence of Mowat-Wilson syndrome in siblings with a novel mutation in the ZEB2 gene. Am J Med Genet A. 2008 Dec 1;146A(23):3095–9.
50. Garavelli L, Cerruti-Mainardi P, Virdis R, Pedori S, Pastore G, Godi M, et al. Genitourinary anomalies in Mowat-Wilson syndrome with deletion/mutation in the zinc finger homeo box 1B gene (ZFHX1B). Report of three Italian cases with hypospadias and review. Horm Res. 2005;63(4):187–92.
51. Cerruti Mainardi P, Pastore G, Zweier C, Rauch A. Mowat-Wilson syndrome and mutation in the zinc finger homeo box 1B gene: a well defined clinical entity. J Med Genet. 2004 Feb;41(2):e16.

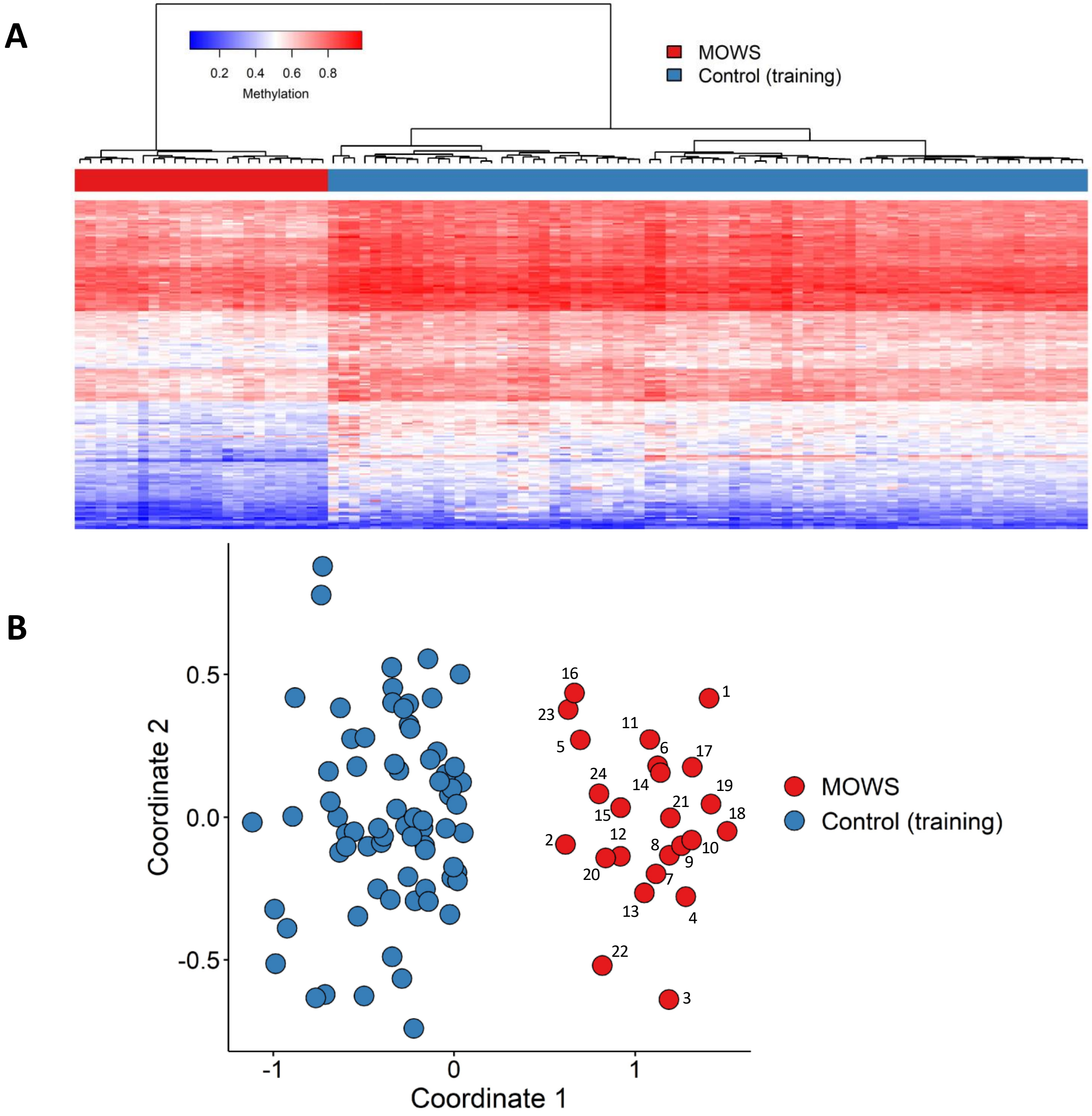

Supplementary figure 1; Mowat-Wilson (MOWS; *ZEB2*) episignature discovery cohort. **(A)** Euclidean hierarchical clustering heatmap, each column represent one MOWS discovery case or control, each row represents one probe selected for this episignature. The analysis shows a clear separation between the cases in red and controls in blue. **(B)** Multidimensional scaling (MDS) plot shows segregation of MOWS cases and controls.

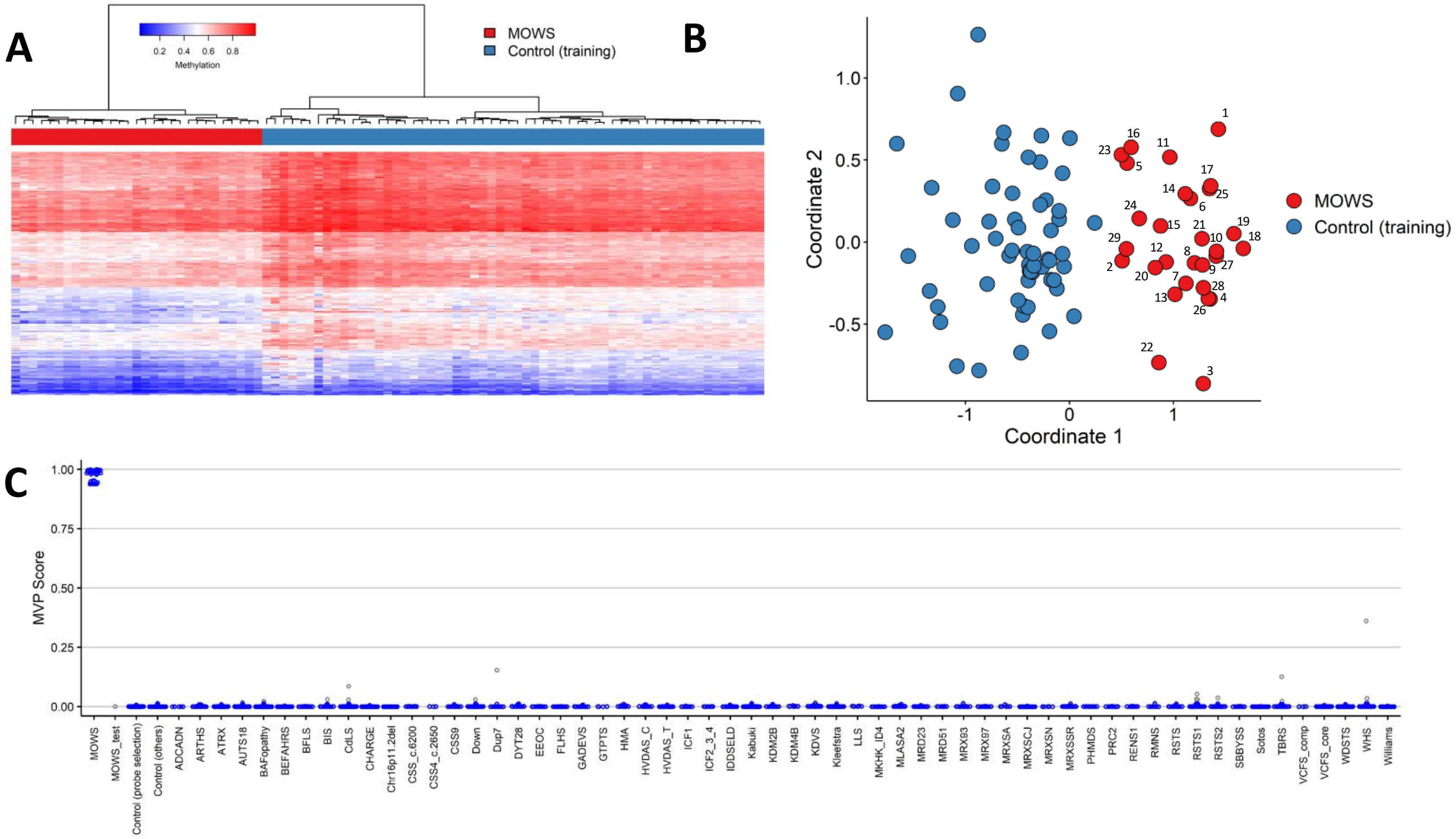

Supplementary figure 2; Mowat-Wilson (MOWS; *ZEB2*) episignature discovery + validation cohort both added as training. **(A)** Euclidean hierarchical clustering heatmap, each column represent one MOWS case or control, each row represents one probe selected for this episignature. It shows a clear separation between the cases in red and controls in blue. **(B)** Multidimensional scaling (MDS) plot shows segregation of MOWS cases and controls. **(C)** Support Vector Machine (SVM) classifier model. Model was trained using the selected MOWS episignature probes, 75% of controls and 75% of other neurodevelopmental disorder samples (blue). The remaining 25% controls and 25% of other disorder samples were used for testing (grey). Plot shows that the MOWS samples all have methylation variant pathogenicity (MVP) scores close to 1.

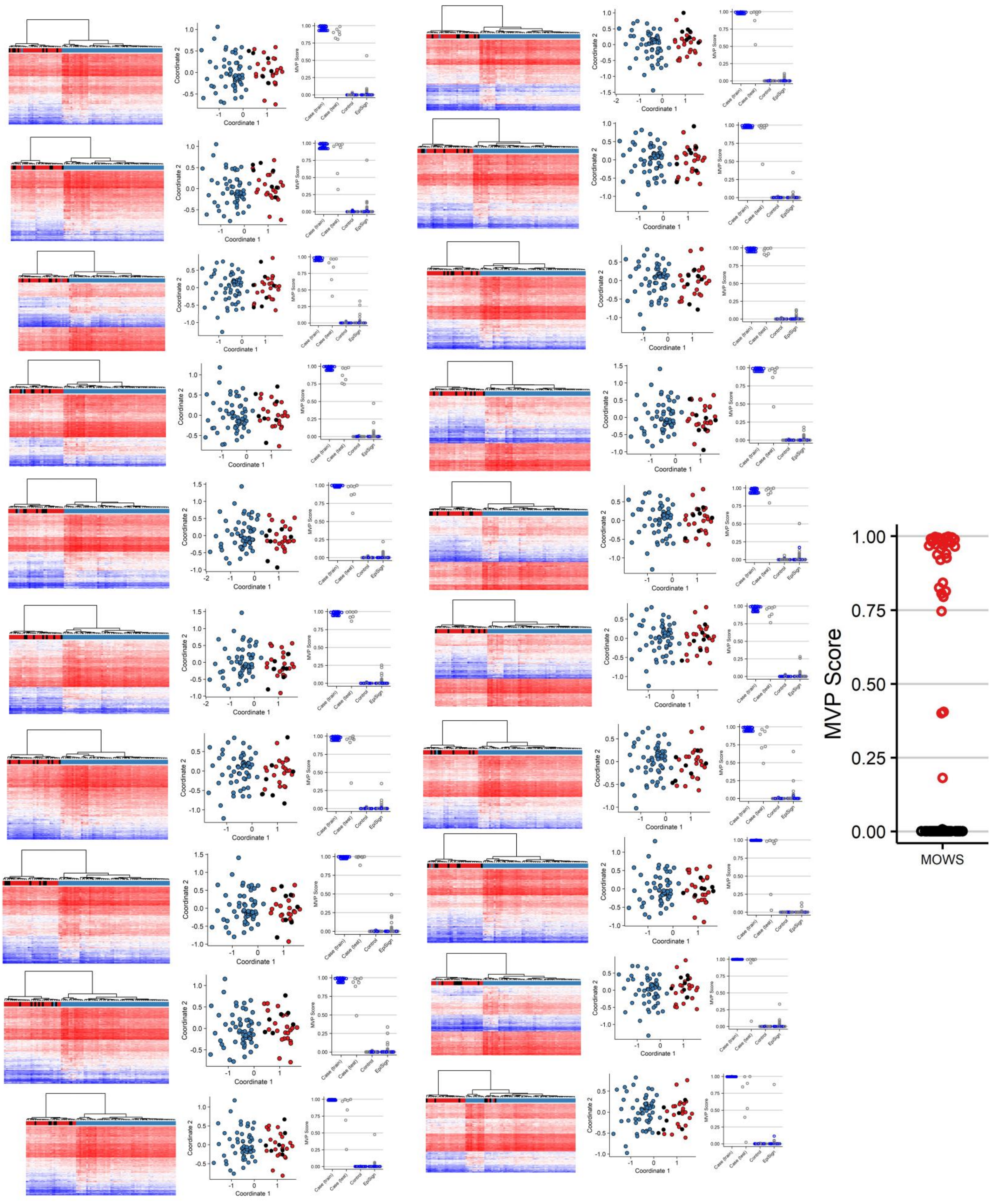

Supplementary Figure 3; 20 rounds of leave-25%-out cross validation, discovery + validation cohort. In each cross-validation set, 7 test case samples (black) are used as testing. The other MOWS cases used for episignature training are shown in red and control training sample shown in blue in the heatmap and MDS plots. The last plots demonstrate the MVP scores of the Support Vector Machine (SVM) classifier model that was trained using the selected MOWS episignature probes from training cases, 75% of controls and other EpiSign samples (blue). The remaining 25% of controls and other disorder samples were used as testing alongside the MOWS cases (grey).

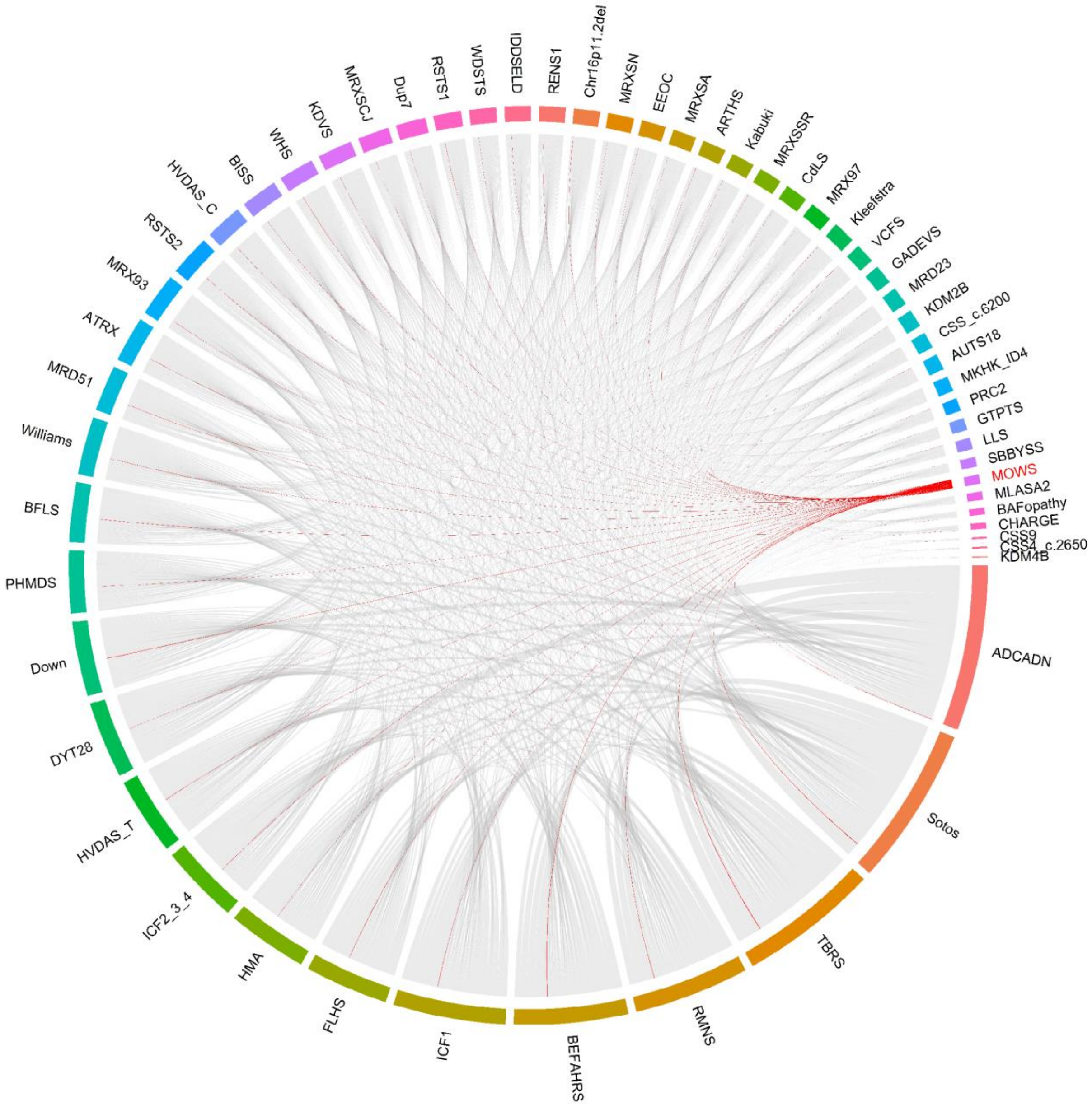

Supplementary Figure 4. Differentially methylated probes (DMPs) shared between the MOWS cohort and 56 other episignatures on EpiSign™. Circos plot representing the probes shared between each pair of cohorts. The thickness of the connecting lines indicates the number of probes shared between the two cohorts. MOWS connections are highlighted in red.

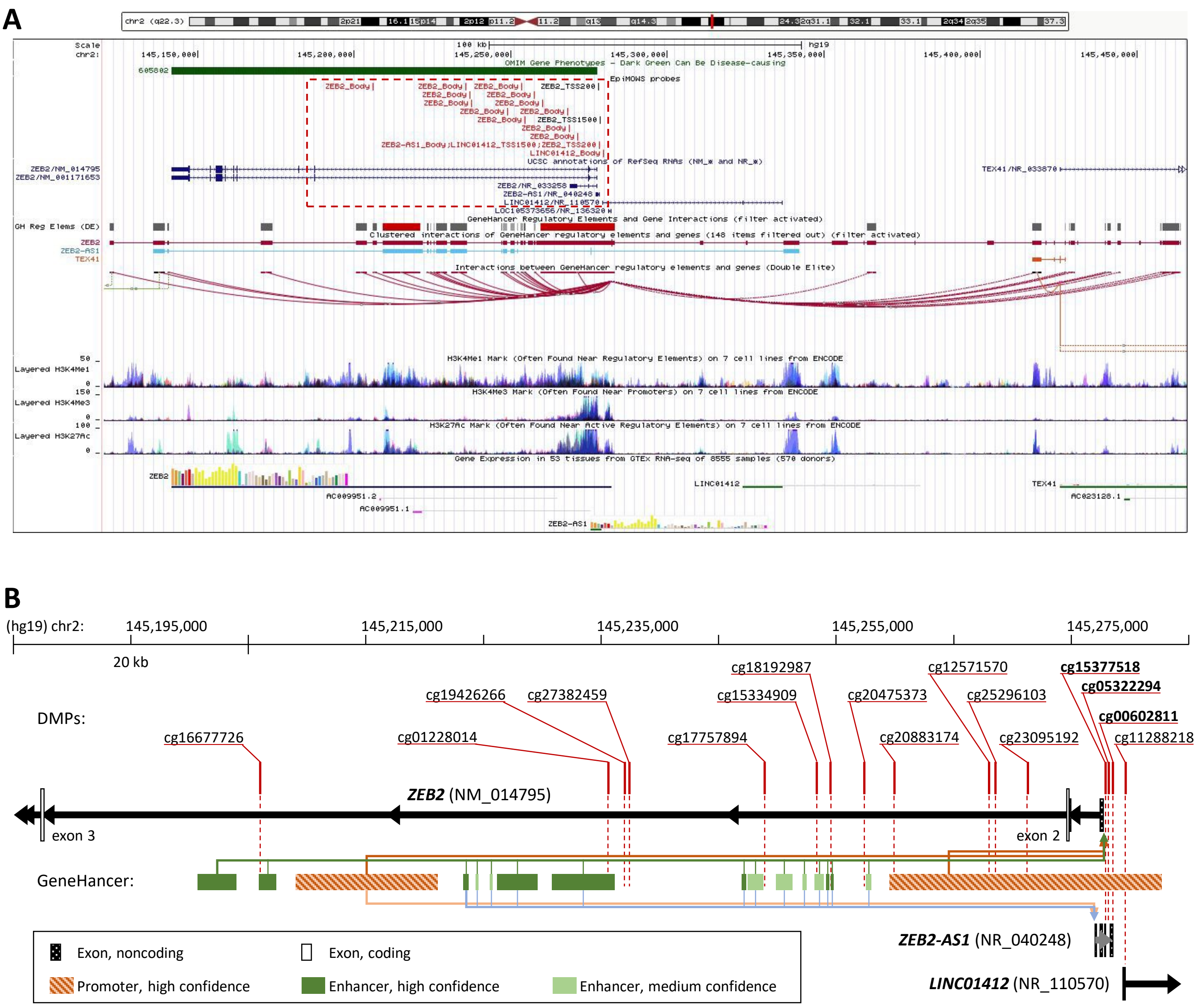

Supplementary Figure 5. Differentially methylated probes (DMPs) from the Mowat-Wilson syndrome DNA methylation profile within the *ZEB2* gene. **(A)** UCSC Genome Browser view (http://genome.ucsc.edu)[a] of Human (GRCh37) chromosome 2 showing the *ZEB2* locus, including its regulatory elements and gene interactions according to the GeneHancer database. The vertical bars in the "EpiMOWS probes" track represent the sixteen DMPs occurring within the *ZEB2* gene, out of the 296 sites that define the episignature. **(B)** Schematic representation of the genomic region encompassing *ZEB2-AS1* and the first three exons of the *ZEB2* gene (dashed-border box in panel A), detailing the relative position of DMPs, transcripts and GeneHancer-annotated regulatory elements. Three DMPs (in bold) map close to the transcription start site (TSS) -200 and -1500 bp positions. All sixteen DMPs are hypomethylated and map to promoter or enhancer regions considered to be involved in the regulation of *ZEB2* transcription.

[a] Kent WJ, Sugnet CW, Furey TS, et al. The human genome browser at UCSC. *Genome Res*. 2002;12(6):996-1006.
